# TNF Inhibits Notch-1 in Skeletal Muscle Cells by Ezh2 and DNA Methylation Mediated Repression: Implications in Duchenne Muscular Dystrophy

**DOI:** 10.1371/journal.pone.0012479

**Published:** 2010-08-30

**Authors:** Swarnali Acharyya, Sudarshana M. Sharma, Alfred S. Cheng, Katherine J. Ladner, Wei He, William Kline, Huating Wang, Michael C. Ostrowski, Tim H. Huang, Denis C. Guttridge

**Affiliations:** 1 Human Cancer Genetics and Department of Molecular Virology, Immunology and Medical Genetics, The Ohio State University, Columbus, Ohio, United States of America; 2 Department of Molecular and Cellular Biochemistry, The Ohio State University, Columbus, Ohio, United States of America; 3 Institute of Digestive Disease and Li Ka Shing Institute of Health Sciences,The Chinese University of Hong Kong, Prince of Wales Hospital, Hong Kong SAR, China; 4 The Arthur G. James Comprehensive Cancer Center, The Ohio State University, Columbus, Ohio, United States of America; Texas A&M University, United States of America

## Abstract

**Background:**

Classical NF-κB signaling functions as a negative regulator of skeletal myogenesis through potentially multiple mechanisms. The inhibitory actions of TNFα on skeletal muscle differentiation are mediated in part through sustained NF-κB activity. In dystrophic muscles, NF-κB activity is compartmentalized to myofibers to inhibit regeneration by limiting the number of myogenic progenitor cells. This regulation coincides with elevated levels of muscle derived TNFα that is also under IKKβ and NF-κB control.

**Methodology/Principal Findings:**

Based on these findings we speculated that in DMD, TNFα secreted from myotubes inhibits regeneration by directly acting on satellite cells. Analysis of several satellite cell regulators revealed that TNFα is capable of inhibiting Notch-1 in satellite cells and C2C12 myoblasts, which was also found to be dependent on NF-κB. Notch-1 inhibition occurred at the mRNA level suggesting a transcriptional repression mechanism. Unlike its classical mode of action, TNFα stimulated the recruitment of Ezh2 and Dnmt-3b to coordinate histone and DNA methylation, respectively. Dnmt-3b recruitment was dependent on Ezh2.

**Conclusions/Significance:**

We propose that in dystrophic muscles, elevated levels of TNFα and NF-κB inhibit the regenerative potential of satellite cells via epigenetic silencing of the Notch-1 gene.

## Introduction

Adult skeletal muscle represents a dynamic tissue in our body that possesses a remarkable capacity to regenerate in response to injury [1], [2]. A normal regenerative response in muscle involves activation and subsequent proliferation of quiescent satellite cells that eventually differentiate and fuse to form new myofibers. However, in pathological states such as muscular dystrophies, satellite cell number as well as their doubling potential are thought to decrease, resulting in impaired regenerative potential [3], [4].

Duchenne muscular dystrophy (DMD) is one such severe myopathy that arises from mutations in the 2.5 Mb DMD gene and is lethal in late childhood due to secondary consequences such as persistent muscle degeneration, exhausted regenerative capacity and respiratory or cardiac failure [5]. The DMD gene codes for a 427 KDa protein, dystrophin that is thought to be critical in maintaining a mechanical as well as signaling link from the extracellular matrix to the cytoskeleton in a muscle cell. The absence of dystrophin in DMD results in progressive muscle weakness, frequent contractures, paucity of regenerating fibers and gradual replacement of muscle fibers with adipose and connective tissue. Despite the advances in understanding the proximate causes of DMD, correcting the primary defect has proven to be a daunting task, with no curative therapy options available against this deadly disease [6].

Although stemming from a single cause, DMD is now increasingly being accepted as multifactorial in progression, with a myriad of signaling pathways influencing its pathological state. The nuclear factor kappa B (NF-κB) pathway represents one such signaling pathway that has been recently linked to both inflammatory responses as well as attenuated regeneration [7], [8]. In mammalian cells, the NF-κB family of transcription factors consists of five subunits namely p65 or RelA, RelB, c-Rel, p50 and p52, which either homo or heterodimerize with each other. In an unstimulated state, NF-κB exists as a latent complex bound to the inhibitor family of proteins or IκB proteins. Upon stimulation with classical inducing signals such as TNFα (from now on simply referred to as TNF) or IL1β, an upstream IκB kinase complex (IKK) is activated, which results in the phosphorylation and degradation of the IκB proteins, releasing the NF-κB dimer to translocate to the nucleus to activate transcription [9], [10]. Depending on the physiological conditions, stimulus and the cell type, the NF-κB signaling pathway maintains a negative feedback loop by activating the transcription of IκBα that re-sequesters the NF-κB complex in the cytoplasm or a positive feedback loop by synthesizing its own inducer, TNF, that can reactivate the pathway [11], [12].

Primarily implicated in immune responses, NF-κB has been also shown to negatively regulate skeletal muscle differentiation [13], [14], [15]. NF-κB is constitutively active in proliferating myoblasts and can inhibit myogenesis by promoting a mitogenic activity via cyclin D1 or by inhibiting the synthesis of MyoD, a bHLH transcription factor that functions in muscle development and repair [16], [17], [18]. More recently, NF-κB was shown to suppress myofibrillar and miRNA-29 gene expression through the regulation of the transcriptional repressor, YY1 [19]. Moreover, treatment of primary myoblasts with the NF-κB inhibitor, curcumin stimulates myoblast fusion thereby enhancing myogenesis [20]. In line with these in vitro findings, activation of the TNF pathway by muscle gene transfer inhibits regeneration in vivo while muscle specific deletion of IKK was recently described to promote secondary myogenesis in response to acute injury signals [21], [22].

Elevated levels of inflammatory mediators such as TNF have been also associated with persistent NF-κB activation under chronic injury conditions in both DMD patients and *mdx* mice [23], [24]. We recently showed that conditional deletion of IKKβ in *mdx* muscle cells decreases TNF expression, which was associated with a concomitant elevation of Pax7^+^, CD34^+^ satellite cells [25]. Since chronic levels of TNF functions as a potent inhibitor of muscle differentiation, it suggests that this cytokine might take part in limiting the regenerative potential of muscle stem cells in dystrophic muscle. Results from this study support this notion, as TNF is able to downregulate Notch-1, a key determinant of satellite cell activation [26]. We find that in vitro, TNF represses Notch-1 in an NF-κB dependent manner. In addition, deletion of IKKβ in *mdx* mice restores Notch-1 levels. Furthermore, distinct from the usual TNF response, we show that TNF repression occurs via a combination of histone and DNA methylation on the Notch-1 promoter. Together, these findings demonstrate a novel activity of TNF for epigenetic regulation of chromatin silencing. We further propose that TNF-mediated repression of Notch functions in dystrophic muscles to limit satellite cell regeneration.

## Results

### TNF inhibits Notch-1 expression

**Figure 1:**
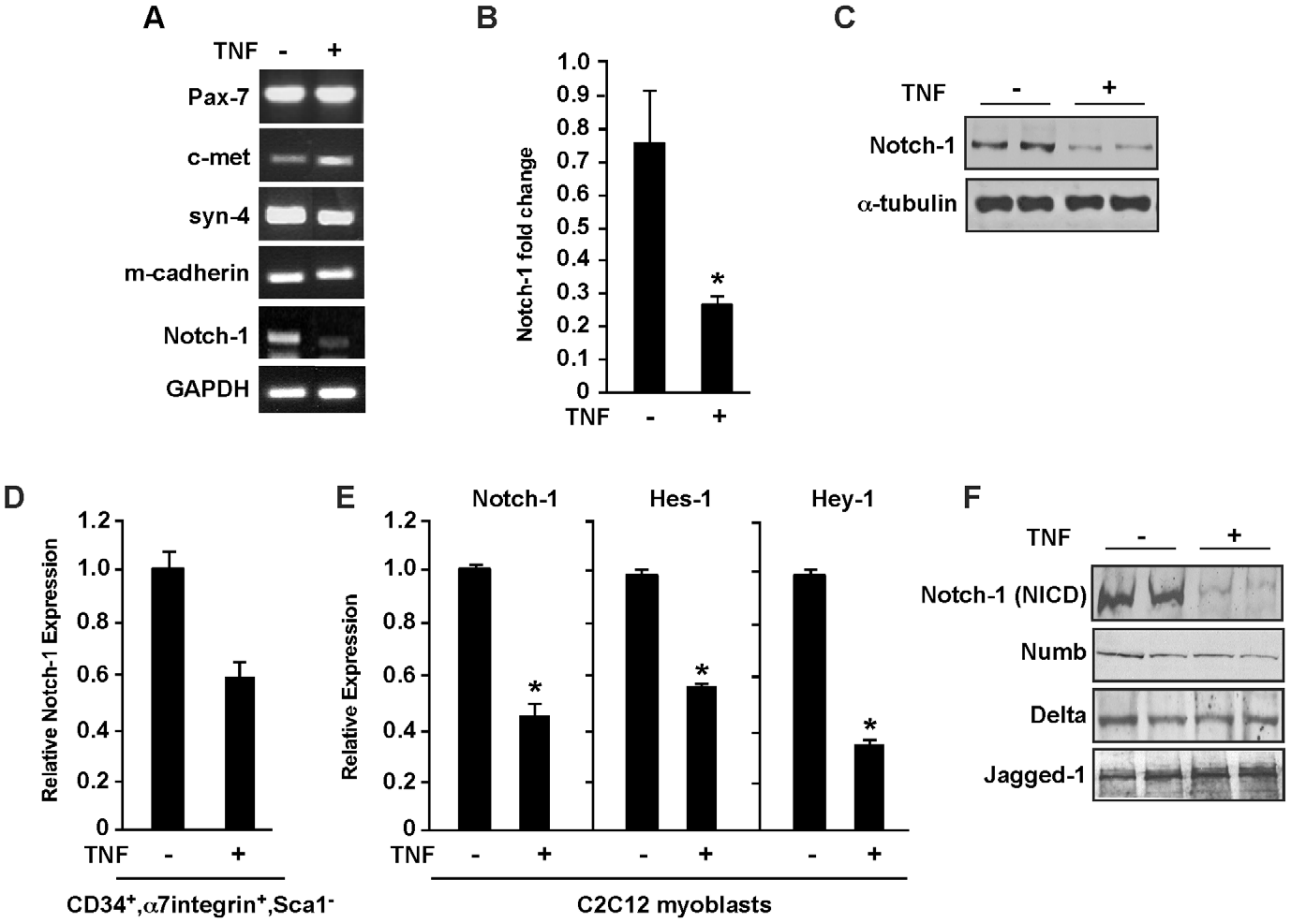
TNF regulates Notch-1 expression. **A.** Mononuclear cell cultures were prepared from *mdx* muscles and cultured under proliferation conditions and either left untreated or treated with TNF. After 24 hrs cells were harvested and processed for total RNA, and subsequently semi-quantitative RT-PCR reactions were performed probing for satellite cell markers. GAPDH was used as a control. **B.** Quantitative real-time RT-PCR analysis of Notch-1 from satellite cell cultures untreated and treated with TNF. Values shown were normalized to GAPDH levels. Asterisk denotes statistical significance, p = 0.03708. **C.** Western blot analysis probing for total Notch-1 receptor from duplicate *mdx* satellite cell cultures treated with TNF. The blot was stripped and re-probed for α-tubulin used as a loading control **D.** Quantitative real-time RT-PCR was repeated as in (B) from FACS sorted mononuclear cells enriched for a CD34^+^, α7integrin^+^, Sca1^−^ population. **E.** C2C12 myoblasts were cultured under proliferating conditions and either not treated or treated with TNF. Total RNA was prepared and quantitative RT-PCR analysis was performed probing for Notch-1 and Notch-1 targets, Hes-1 and Hey-1. Asterisk denotes statistical significance; Notch-1, p = 0.04844; Hes-1, p = 0.001; Hey-1, p = 0.0149. **F.** C2C12 myoblasts were cultured in treated in duplicate with TNF and protein lysates were subsequently probed with the intercellular activated form of Notch-1 (NICD), or with Notch signaling components, Delta and Jagged-1.

Recently, we reported on the role of the IKK/NF-κB signaling pathway in the *mdx* model of muscular dystrophy [25]. Specifically, we demonstrated using mutant mice deficient in skeletal muscle derived IKKβ that NF-κB signaling functions in dystrophic muscle to limit the regenerative potential of muscle satellite cells. We also found that this regulation coincided with elevated levels of TNF, leading us to speculate that TNF production by NF-κB is muscle derived and acts in paracrine fashion to suppress myogenesis of satellite cells. To explore this notion, freshly isolated, pre-plated mononuclear cells from *mdx* muscles were treated with TNF and analyzed for differences in genes Pax7, c-met, syndecan-4, M-cadherin, and Notch-1, which are important regulators of satellite cell specification, biogenesis, migration, and adhesion. Results showed that TNF had no effect on Pax7, c-met, syndecan-4, or m-cadherin (Fig. 1A). In contrast, cytokine treatment caused a significant reduction in Notch-1 expression (Fig. 1A), a result that we confirmed by both quantitative RT-PCR and western blotting (Fig. 1B and 1C). This regulation appeared specific to Notch-1 since its closely related homolog, Notch-2 was unaffected by TNF (data not shown). Since mononuclear cells represent a mixed population, we repeated our analysis with enriched satellite cells by FACS sorting for CD34^+^, α7integrin^+^, Sca1^−^ cells, and again observed in duplicate experiments that Notch-1 was downregulated by TNF (Fig. 1D). In addition, TNF reduced Notch-1 in C2C12 myoblasts (Fig. 1E) suggesting that this regulation is not selective to *mdx* progenitor muscle cells. Moreover, Notch-1 transcriptional target genes, Hes-1 and Hey-1 were also significantly affected (Fig. 1E), further indicating that TNF mediates repression on Notch-1 signaling.

This activity of the Notch signaling is initiated by binding of Notch ligands such as Delta and Jagged to the notch receptor, which in turn leads to an enzymatic cleavage of the receptor and release of the activated Notch-1 protein known as Notch intracellular domain (NICD) into the cytoplasm [27]. NICD then translocates to the nucleus and binds to transcription factors such as RBP-J and Suppressor of Hairless Su(H). The Notch signaling pathway is additionally regulated by increased expression of its inhibitor, Numb that can antagonize the activity of NICD preventing its nuclear translocation [28]. To determine whether TNF affects other components of Notch signaling, we examined the levels of Notch-1 and its activated form, NICD. As predicted from the downregulation of Notch-1 mRNA, and total Notch receptor protein (Fig 1A and 1C), the NICD form of Notch-1 was also strongly downregulated by TNF in C2C12 myoblasts (Fig. 1F). However, the levels of the ligands, Delta and Jagged as well as the Notch inhibitor, Numb remained unchanged, indicating that TNF mediated repression of Notch-1 is independent of its ligands or inhibitor levels.

**Figure 2:**
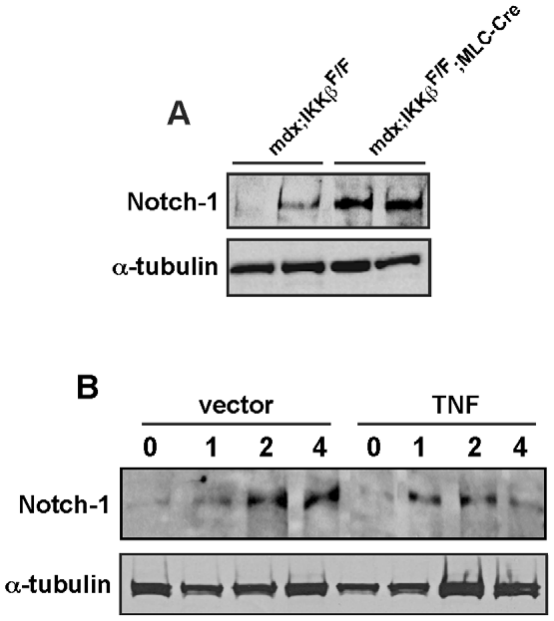
TNF regulates Notch-1 expression in vivo. **A.** Muscle homogenates were prepared from 7–8 week old *mdx* mice that either contained or lacked IKKβ and western analysis was performed probing for Notch-1. The blot was stripped and reprobed for α-tubulin used as a loading control. **B.** Nude mice were implanted with vector control CHO cells or CHO cells expressing TNF. Once tumors were established, muscle injury was induced to tibialis anterior muscles with cardiotoxin injections. At indicated days post-toxin injections, muscles were harvested and homogenates prepared for western analysis probing for Notch-1 and α-tubulin as a loading control.

Next, we examined Notch-1 levels in IKKβ deleted *mdx* muscles which were found to express less TNFα [25]. Results showed that Notch-1 was increased in these muscles compared to *mdx* IKKβ flox control littermates (Fig. 2A). To further evaluate the effect of TNF on Notch-1 in the context of muscle regeneration, tibialis anterior muscles were injured with cardiotoxin injection in mice that had previously been implanted subcutaneously with vector control or TNF secreting Chinese hamster ovary (CHO) cells that developed as a localized tumor [29]. Whereas cardiotoxin induced Notch-1 in control mice at levels that persisted over 4 days, this regulation was noticeably blunted in mice expressing TNF (Fig. 2B). Together these data are suggestive that Notch-1 is a target of TNF in vivo.

### TNF requires NF-κB to down regulate Notch-1

**Figure 3:**
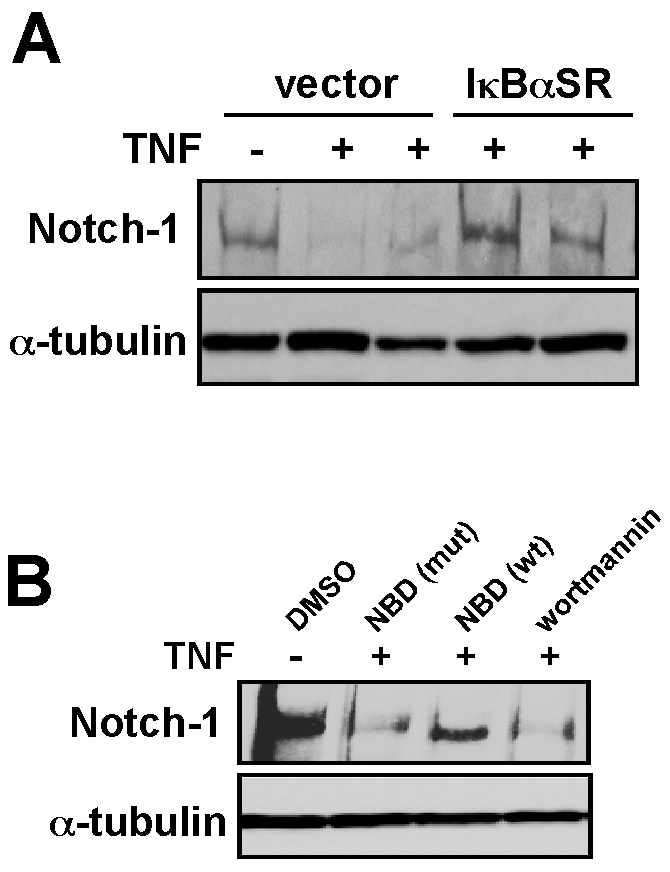
TNF regulation of Notch-1 is dependent on NF-κB. **A.** Western analysis of Notch-1 was performed in either untreated or TNF treated C2C12 vector myoblasts or myoblasts stably expressing the IκBα-SR transgene. **B.** C2C12 myoblasts were pre-treated for 1 hr with either DMSO, an IKK inhibitor peptide (NBD) in both a wild type (wt) and inactive mutant form (mut), or with a treatment of wortmannin. Following TNF treatment, cells were harvested and western was performed probing for Notch-1 and α-tubulin as a control.

Although TNF is a pleiotropic factor, much of its signaling potential occurs through pathways like NF-κB [9]. We therefore asked whether NF-κB activity was required for TNF mediated regulation of Notch-1. Results showed that compared to control treated myoblasts, cells inhibited in NF-κB through expression of the degradation resistant IκBα-SR mutant retained Notch-1 expression upon TNF treatment (Fig. 3A). To confirm this regulation, C2C12 myoblasts were treated with TNF in the presence of the specific inhibitor of the IKK complex, the Nemo Binding Domain (NBD) peptide [30]. Results showed that wild type NBD, but not mutant peptide or a PI3K/Akt inhibitor (wortmannin), blocked Notch-1 suppression by TNF (Fig. 3B). These data support the requirement of NF-κB in TNF-mediated repression of Notch-1.

### Notch-1 repression by TNF is mediated by recruitment of Ezh2

**Figure 4:**
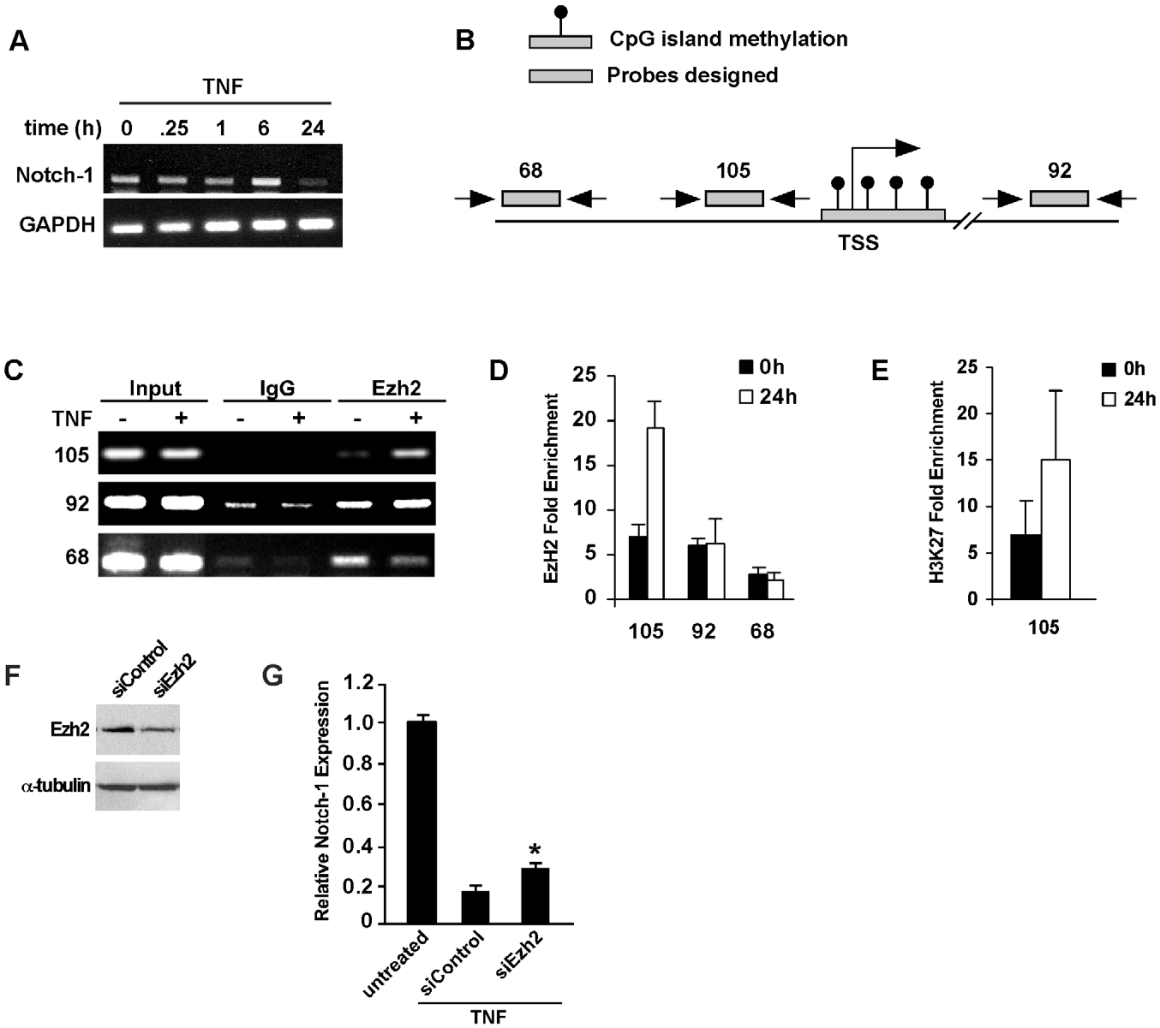
TNF repression of Notch-1 occurs through the recruitment of Ezh2. **A.** Time course analysis of Notch-1 expression following TNF treatment of C2C12 myoblasts. **B.** Schematic illustration of the Notch-1 gene indicating conserved regions of CpG content (blue bars) that are located in positions proximal to the transcriptional start site (TSS), and where probes were designed for ChIP analysis. Also indicated is a CpG island immediately surrounding the TSS (red bar). **C, D.** C2C12 myoblasts were treated with TNF and at indicated times cells were harvested and subsequently prepared for ChIP analysis probing for Ezh2 by both semi-quantitative (**C**) and quantitative (**D**) RT-PCR. **E.** Using cell extracts prepared in C, additional ChIPs were performed for methylation of H3K27. F. C2C12 myoblasts were transfected with scrambled siRNA (siControl) or siRNA targeting Ezh2 (siEzh2) and following 48 hrs, western blot analyze was performed probing for Ezh2 and α-tubulin used as a loading control. G. C2C12 myoblasts transfections were performed with siControl and siEzh2 oligonucleotides and next day cells were treated for TNF for 48 hrs, and subsequently processed for Notch-1 expression by real-time RT-PCR. Asterisks denotes p = 0.00314).

To gain further insight into the mechanism of Notch-1 regulation, C2C12 myoblasts were treated with TNF and the kinetics of Notch-1 expression was examined. Results revealed that Notch reduction occurred slowly reaching maximum suppression by 24 hrs (Fig. 4A). Given that NF-κB activation in response to TNF occurs normally within minutes, it suggested that another mechanism indirect of NF-**κ**B might be involved. This led us to investigate whether co-repressor complexes could act in the transcriptional silencing of Notch-1. One such co-repressor complex that was explored was the histone deacetylases (HDACs) that are typically found associated with mSin3 or NuRD and are often recruited to promoters to silence their expression [31], [32]. However, pretreatment of C2C12 myoblasts with the HDAC inhibitor, Trichostatin A at different doses did not restore Notch-1 levels in the presence of TNF (data not shown). In addition, chromatin immunoprecipitation assay (ChIP) with HDAC-1, mSin3 or Mi2 antibodies at three different regions of the Notch-1 promoter did not show any differences in binding upon TNF treatment (data not shown), arguing against the possibility of histone deacetylase involvement.

Another repressor complex regulating transcriptional silencing is the Polycomb Group (PcG), composed of proteins such as Enhancer of Zeste homolog 2 (Ezh2), Suppressor of Zeste12 and embryonic ectoderm development [33]. Histone methylation is thought to be one of the primary mechanisms of transcriptional repression by the PcG as shown in the case of inactivation of the X-chromosome. To access the role of the PcG in Notch-1 regulation, we performed a bioinformatic analysis of the Notch-1 promoter to delineate regions of interest based on their conservation, CpG content, and position relative to the transcription start site (TSS). Three regions were chosen including a conserved site approximately 1 kb upstream of the TSS (designated region 68), a second region adjacent to a CpG rich area near the TSS (designated 105) and a third region approximately 10 kb downstream of the TSS (designated 92) (Fig. 4B). Next, we performed ChIP analysis with Ezh2 followed by probe-based PCR, using primers amplifying the regions of interest. Both semi- and quantitative amplification revealed that TNF treatment stimulated Ezh2 association with region 105 that maps specifically between nucleotides −309 to −223 of the Notch-1 promoter (Fig. 4C and 4D). This association was time dependent and selective, since Ezh2 binding was maximal at 24 hrs and not observed with regions 92 or 68. Interestingly, although NF-**κ**B has been shown to regulate YY1 to silence myofibrillar genes, such as troponin and myosin isoforms and miR-29 in progenitor myoblasts [19], ChIP analysis in response to TNF found that this PcG associated protein was not recruited with Ezh2, nor were we able to find YY1 binding sites within this region of the Notch-1 promoter (data not shown).

Since Ezh2 is a methyltransferase that has high binding specificity for lysine 27 in histone H3 (H3K27) [34], [35], [36], we asked whether Ezh2 binding to the Notch-1 promoter translated to its functional activity. ChIPs performed on region 105 detected an increase in H3K27 methylation after 24 hrs of TNF treatment (Fig. 4E). Significantly, this methylation coincided with Ezh2 binding and Notch-1 downregulation. To test the requirement of Ezh2 for TNF-mediated repression of Notch-1, we transfected C2C12 myoblasts with siRNA targeting Ezh2. Western blotting confirmed the efficiency of Ezh2 knockdown (Fig. 4F), and compared to scrambled siRNA control, silencing of Ezh2 restored Notch-1 expression in the presence of TNF (Fig. 4G). Although the level of Notch-1 restoration was admittedly modest, this most likely reflects the degree to which Ezh2 silencing was achieved by transfection of siRNA oligonucleotides. Taken together, these results suggest that in response to TNF, Ezh2 is recruited to the Notch-1 promoter, which catalyzes the addition of methyl groups to H3K27 and directs a repressed chromatin state resulting in the downregulation of Notch.

### TNF promotes DNA methylation on the Notch-1 promoter

**Figure 5:**
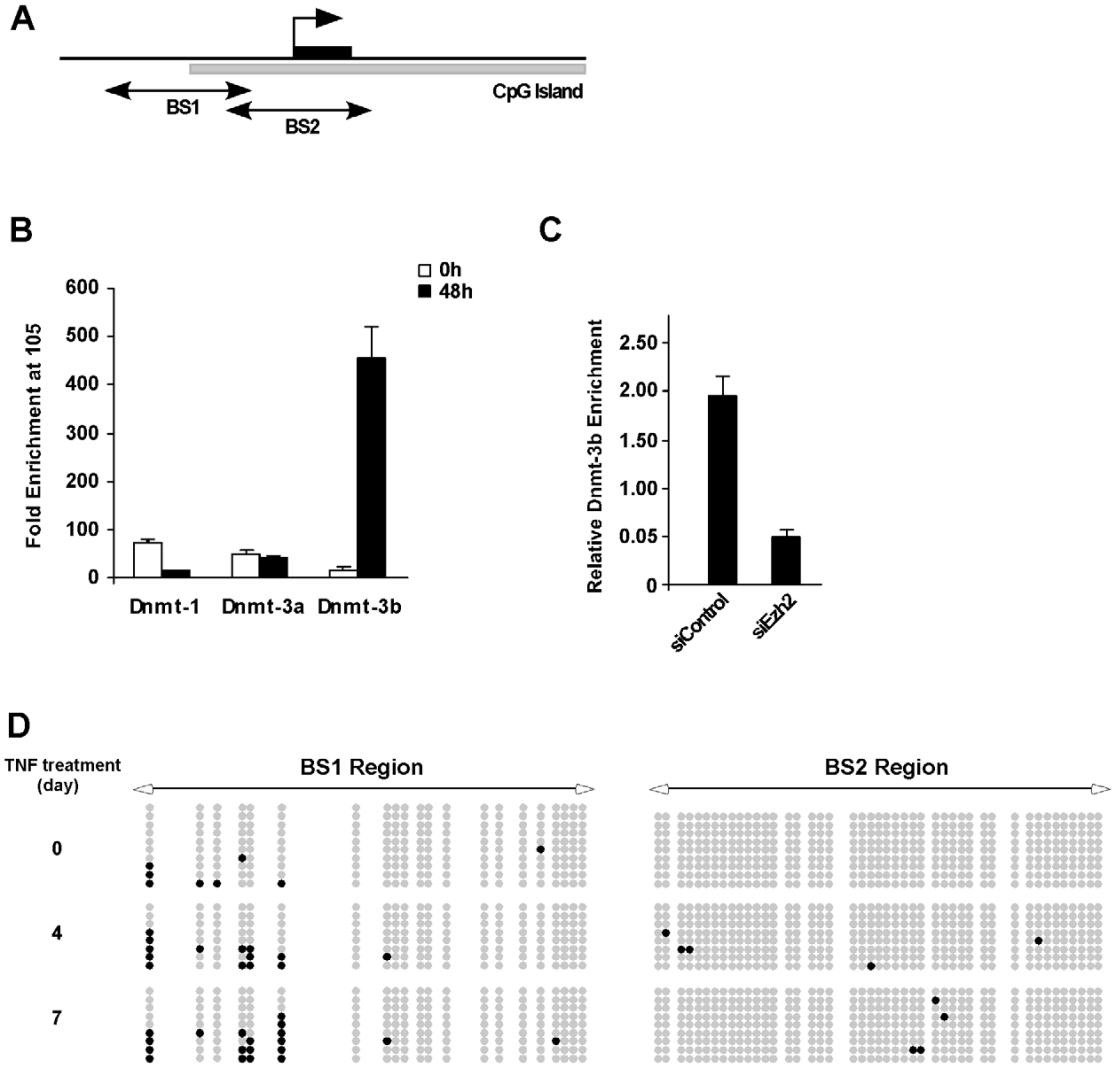
TNF promotes Dnmt-3b recruitment and DNA methylation on the Notch-1 promoter. **A.** Schematic illustration of the Notch-1 promoter indicating the CpG islands in the BS1 and BS2 regions proximal to the TSS. **B.** C2C12 myoblasts were treated with TNF for 48 hrs at which time cells were prepared for quantitative ChIP analysis for Dnmt-1, Dnmt-3a, and Dnmt-3b. **C.** C2C12 myoblasts were transfected with scrambled siRNA or siRNA against Ezh2 and the following day cells were treated for TNF for 48 hrs and ChIP analysis for Dnmt-3b was subsequently performed. **D.** C2C12 myoblasts were treated with TNF for up to 7 days and at indicated times, cells were processed for bisulfite sequencing of the BS1 region within the Notch-1 promoter. Note the increase in BS1 methylation at discrete CpG dinucleotides over time with TNF treatment.

Histone methylation is often thought to act as a recruitment platform for DNA methylation to occur [37]. Therefore, in the final part of the study, we addressed whether Notch-1 promoter silencing by TNF also involved methylation of CpG dinucleotides. Bisulfite sequencing was performed on the Notch-1 promoter surrounding the Notch-1 TSS, but outside region 105 (designated region BS2, Fig. 5A), in TNF treated myoblasts. However, at 24 and 48 hrs no evidence of DNA methylation was detected within the TSS (data not shown). We therefore asked whether methylation could occur in a more selective region. In support of this notion, we detected a smaller CpG island (BS1) within region 105, which coincided with Ezh2 binding. ChIP analysis further revealed that methyltransferase Dnmt-3b, but not Dnmt1 or 3a, was strongly recruited to region 105 following 48 hrs of TNF treatment (Fig. 5B). Dnmt-3b recruitment was found to be dependent on Ezh2, since siRNA silencing of Ezh2 prior to the addition of TNF led to a significant reduction of Dnmt-3b binding to region 105 of the Notch-1 promoter (Fig. 5C). Together, these data indicated that DNA methylation most likely occurs within the smaller BS1 CpG island. To confirm this possibility, C2C12 myoblasts were treated with TNF and bisulfite sequencing was repeated on the BS1 region. Significantly, an increase in methylation was detected in BS1 following TNF treatment. This regulation appeared specific since methylation started after 4 days of treatment, and occurred at discrete residues within the BS1 region (Fig. 5D). We infer from these results that Notch-1 silencing occurs from a combination of Ezh2 and Dnmt-3b epigenetic regulation. Whereas Ezh2 histone methylation occurs within the first 48 hrs, long-term suppression of Notch-1 by TNF is maintained through Dnmt-3b methylation of BS1 in the Notch-1 promoter that encompasses the same 105 region of PcG binding. Thus, this may be one mechanism through which TNF is capable of inhibiting Notch-1 expression and satellite cell activation in *mdx* mice.

## Discussion

Satellite cells and their descendant myoblast population remain as a potential source of regeneration that can be harnessed therapeutically in life threatening degenerative diseases such as DMD [4]. As a step in furthering our understanding of satellite cell regulation, this study explored how TNF in the muscle environment represses the satellite cell program. The Notch signaling pathway specifically plays a crucial role in satellite cell activation, proliferation and self-renewal [26]. In line with studies showing that the inhibition of the TNF signaling pathway is beneficial in DMD [21], [38], [39], [40], our findings elucidate a new mode of action of TNF in downregulating Notch-1 that may be relevant in muscular dystrophies.

Contrary to the most well studied mechanism of TNF mediated transcriptional activation via NF-**κ**B or c-jun/AP1 [9], [10], this study identified Notch-1 as one of the few targets that can instead be transcriptionally repressed in response to TNF. Our study provides first time evidence that TNF silencing of Notch-1 in muscle cells occurs epigenetically, by a concerted recruitment of histone and DNA methyltransferases. TNF-mediated repression of Notch-1 also appeared to require NF-**κ**B activation. How NF-**κ**B is involved in regulating epigenetic repression of the Notch-1 promoter is not known at this time, yet several recent mechanisms have been described to support a role for NF-**κ**B in gene silencing. In the presence of UV radiation and certain chemotherapeutic drugs, NF-**κ**B has been shown to repress anti-apoptotic genes such as Bcl-XL and A20 by associating with histone deacetylases [41], and in the context of myogenesis, our group has recently shown that NF-**κ**B can indirectly repress troponin-I2 and miR-29 by transcriptionally activating the polycomb repressor, YY1 [19]. In addition, based on work by Ndlovu and colleagues [42], NF-**κ**B was shown to increase chromatin accessibility across the IL-6 promoter in breast cancer cells. Hence, it can be speculated that increased chromatin accessibility mediated by NF-**κ**B can facilitate binding of histone and DNA methylation complexes under certain conditions.

Evidence exists in favor of histone and DNA methylation preceding one another in diverse cell types and conditions [43], [44]. In fact, gene silencing in many instances can also occur by either histone or DNA methylation by mutually exclusive mechanisms [45], [46]. However, findings from the current study suggest a sequential recruitment of the polycomb associated histone methyltransferase, Ezh2, followed by DNA methyltransferase, Dnmt-3b, to the notch promoter in response to TNF. A similar cooperative relationship between the polycomb repressors, Ezh2, Suppressor of Zeste12, and DNA methyltransferases has been shown in HOXA9 gene silencing upon loss of p16^INK4a^ tumor suppressor activity in breast cancer cells [47]. However, unlike the HOXA9 locus, de-novo methylation on the Notch-1 promoter is not as extensive and is restricted only to a few bases. The potential reason behind selective methylation of the notch promoter in spite of significant recruitment of Dnmt-3b upon TNF treatment is currently unknown. Nonetheless, in favor of minimal methylation mediating transcriptional repression, a few single sites of the p53 and rDNA promoters when methylated can effectively suppress gene expression [48], [49], and interestingly, Dnmt-3b was associated with methylation of discrete cytosine in non-CpG islands of the peroxisome proliferator-activated receptor γ coactivator-1α promoter (50). Moreover, Ezh2 was also shown to directly interact with DNMTs to repress certain genes such as MYT1 [37]. Whether a similar physical association can occur in muscle satellite cells during TNF mediated repression of Notch-1 remains to be elucidated.

In summary, based on our previous study [25] and current findings, we envision that the NF-**κ**B pathway functions to limit muscle regeneration at multiple levels in the context of DMD. Conditional deletion of the IKK/NF-**κ**B pathway in mature myofibers in *mdx* mice results in a reduction in TNF and an increase in the number of satellite cells. One mechanism by which TNF has been shown to be relevant in DMD is by promoting muscle necrosis potentially through its inflammatory activity [38], [50]. We propose that an additional role for TNF in DMD is to negatively regulate satellite cell activation through Notch-1 suppression. Interestingly, this TNF-mediated downregulation of Notch levels is NF-**κ**B dependent and additionally involves epigenetic silencing by a combination of histone and DNA methylation. Finally, given the relevance of TNF in several pathophysiological states such as cancer and aging [51], [52], and its connection with the deregulation of the Notch signaling pathway implicated in these diseases [26], [53], [54], suggests that the significance of TNF regulation of Notch-1 may extend to tissues other than skeletal muscle.

## Materials and Methods

### Cell culture

C2C12 cells were obtained from ATCC and cultured and differentiated as described [17]. Generation of C2C12 myoblasts stably expressing a wild type or mutated NF-κB luciferase reporter and cells expressing the IκB-SR (super-repressor) transgene have been previously described [16]. Transient transfections and luciferase assays on C2C12 cells were performed as previously described [19]. siRNA sequences targeting Ezh2 were transfected as mixture using Lipofectamine 2000 following manufacturer's instructions [19]. Satellite cell and myoblast cultures were prepared from whole muscles by isolating mononuclear cells from 2-day old neonates and performing pre-plating steps to remove contaminating fibroblasts or by FACS sorting as described [25], [55], [56], [57].

### Materials

Antibodies against total Notch-1, the intracellular domain of Notch, and Delta were purchased from Santa Cruz Biotechnology. Antibody for α-tubulin was obtained from Sigma, and for Numb and Jagged from the Developmental Hybridoma Bank at Iowa. For ChIP analysis, antibodies against mSin3A, histone deacetylase 1 (HDAC1), YY1 and Mi-2 were purchased from Santa Cruz Biotechnology. Antibodies against Ezh2, and trimethyl-H3K27 were purchased from Cell Signaling, Zymed, and Upstate, respectively. Antibodies against Dnmt-1, Dnmt-3a and Dnmt-3b were purchased from Imgenex. Isotype immunoglobulin G (IgG; Sigma) was used as negative control in ChIP analysis. For FACS, CD34-Alexa Fluor 647 (Clone RAM34) was obtained from Ebioscience, Ly-6A/E (Sca-1)-PE from BD Pharmingen, and α7-integrin-FITC from MBL International. Human TNF was purchased from Promega. Pharmacological inhibitors of NF-κB and PI3K/Akt were purchased from Calbiochem and used as described [58], [59]. Wild type and mutant NBD peptides were a kind gift from AS Baldwin (University Of North Carolina, Chapel Hill). Trichostatin A was purchased from ICN Biomedical, dissolved in ethanol and used as described [46]. Ezh2 Stealth RNAi, set of 3 (ID no. MSS204098, MSS204099, and MSS204100), were obtained from Invitrogen.

### RNA and protein analysis

For RNA expression quantification, 400 ng of total purified RNA was reverse transcribed using the first-strand synthesis system (Roche), or for lower yields of RNA obtained from cell sorting, cDNA synthesis was performed with the iScript kit (BioRad). cDNA corresponding to approximately 5 ng of initial RNA was next used for each quadruplicate quantitative PCR reaction. Mouse Notch-1, Hey-1, Hes-1, and β-actin (as endogenous control) were amplified using commercially designed inventoried Taqman gene expression assays and Taqman Universal PCR master mix (Applied Biosystems). Quantitative RNA expression data were acquired and analyzed in 384-well-plate format using an ABI Prism 7900HT Sequence Detection System (Applied Biosystems) relative to endogenous control. For western blot analysis, extracts were prepared in the presence of protease inhibitors as previously described [16], and results are representative of experiments repeated at a minimum of three times.

### Cell sorting

For FACS, limb muscles from 6-week old *mdx* mice were minced and digested in PBS collagenase and dispase at 37°C with agitation. DMEM-H with 10% heat activated horse serum was used to stop enzymatic digestion and two sequential filtrations with 70 µm and 40 µm screens were performed. Cell pellets were then resuspended in cold PBS containing 0.5% BSA and incubated with primary antibodies for 30 min on ice. Flow cytometry analysis and cell sorting were performed on a FACS Aria (Becton Dickingson), with appropriate isotype matching controls. Sorted CD34^+^, α7integrin^+^, Sca1^−^ cells were then immediately placed in culture with F10 medium containing 20% FBS and fibroblast growth factor supplementation with or without TNF treatment for 24 hrs.

### Mice

Mdx (C57/Bl/10 ScSn DMD^mdx^) were purchased from The Jackson Laboratory. IKK floxed mice and MLC-Cre mice were used as previously described [60], [61] to generate *mdx* IKK floxed and *mdx* IKK floxed- MLC cre mice. All the genotypes were determined by PCR analysis from tail DNA. Mice were housed in the animal facilities of The Ohio State University Comprehensive Cancer Center under conventional conditions with constant temperature and humidity and fed a standard diet. All animal experimentation were approved by The Ohio State University Animal Care and Use Committee. For cardiotoxin experiments, 100 µl of 10 µM cardiotoxin dissolved in PBS was injected into the TA of mice previously injected with CHO cells overexpressing either an empty vector or TNF [17].

### ChIP analysis

Chromatin immunoprecipitation were performed as described [62]. Briefly, cells were cross-linked and soluble chromatin with an average size of 200–1000 bp were prepared by sonication. Pre-cleared soluble chromatin was incubated with 5 µg of antibodies. Immunoprecipitated DNA-protein complexes were washed extensively, eluted and de-crosslinked. Precipitated DNA was further purified with PCR purification according to manufacturer's instructions (Qiagen). Samples were analyzed by real-time PCR by the Universal Probe library (Roche) using the Faststart Taqman Master kit (Roche) for the Notch-1 promoter. The threshold for the promoter was adjusted by that of input values and represented as relative enrichment. All quantitative PCRs were analyzed by melting curve analysis and agarose gels to confirm the presence of a specific single band. Data presented are representative of assays repeated a minimum of three times.

### Bisulfite sequencing

Bisulfite sequencing was performed as described in [63]. Briefly, DNA was isolated from C2C12 myoblasts using QIAmp DNA minikit and 0.5 µg from each sample was treated with sodium bisulfite using EZ DNA Methylation kit (ZYMO Research). Primer sequences were designed using Methyl Primer Express Software v1.0 (Applied biosystems). Primer sequences and amplification conditions are available upon request. Amplified products were electrophoresed on 2% agarose gels and visualized with ethidium bromide. PCR products were cloned into TOPO TA cloning kit (Invitrogen). Six to ten randomly picked clones were sequenced using ABI 3730 DNA analyzer (Applied Biosystems) and analyzed using the BiQ Analyzer.

### Statistical Analysis

All quantitative data are represented as mean ± SEM unless mentioned. Analysis was performed between different groups using 2-tailed student's t-test and nonparametric Mann-Whitney U test. Statistical significance was set at a value of p<0.05.
